# Kyste hydatique cérébral chez l'enfant: à propos de 5 cas

**DOI:** 10.11604/pamj.2014.17.149.3419

**Published:** 2014-03-03

**Authors:** Mohamed Belahcen, Khalid Khattala, Aziz Elmadi, Youssef Bouabdellah

**Affiliations:** 1 Service de chirurgie pédiatrique faculté de médecine université Mohamed 1 Oujda Maroc; 2 Service de chirurgie pédiatrique CHU Hassan 2 Fès, Maroc

**Keywords:** Kyste hydatique, cerveau, enfant, hydatid cyst, brain, child

## Abstract

Le kyste hydatique cervical est une pathologie rare, mais non exceptionnel chez l'enfant. Nous rapportons rétrospectivement une série de cinq cas de kyste hydatique cérébral opérés, avec une revue de la littérature. Le syndrome d'hypertension intracrânien a été révélateur dans la majorité des cas. Le diagnostic a été posé par la TDM cérébrale, le traitement a été chirurgical dans tout les cas, avec une rupture du kyste dans un seul cas, traité par l'albendazol en post opératoire. L’évolution a été bonne dans 3 cas, dans un cas l'atrophie optique était irréversible, et dans un autre cas l'enfant a présenté un syndrome maniaque stabilisé sous traitement. En conclusion le kyste hydatique cérébral reste une cause non négligeable de manifestations neurologiques dans les pays endémiques, le diagnostic positif est fait par la TDM, le traitement est chirurgical, et le pronostic est généralement bon.

## Introduction

La pathologie hydatique est très fréquente au Maroc et au pourtour méditerranéen [1]. La localisation cérébrale est rare, ne représente que 2% environ de toutes les localisations hydatiques de l'organisme et touche surtout l'enfant et l'adolescent [2]. Les signes cliniques sont ceux d'un processus intracérébral, la TDM souvent suffisante pour poser le diagnostic, et le traitement reste chirurgical

## Méthodes

Nous avons conduit une étude rétrospective, étendue sur 03 ans, portant sur les dossiers medicaux de 5 cas de kystes hydatiques cérébraux opérés au service de chirurgie pédiatrique au CHU Hassan 2 Fès-Maroc depuis janvier 2009 jusqu'au décembre 2011.

## Résultats

**Figure 1:**
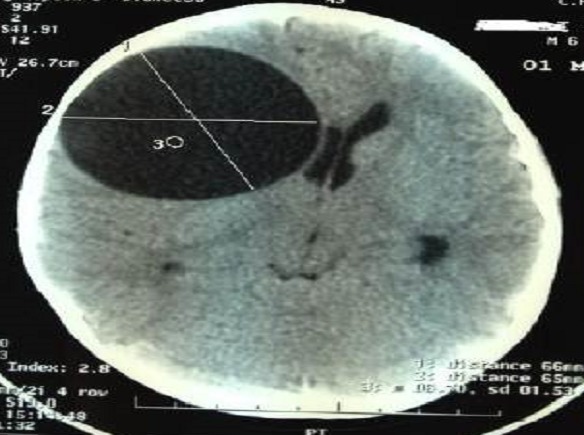
TDM d'un kyste hydatique cérébral de siège fronto-pariétal droit

**Figure 2:**
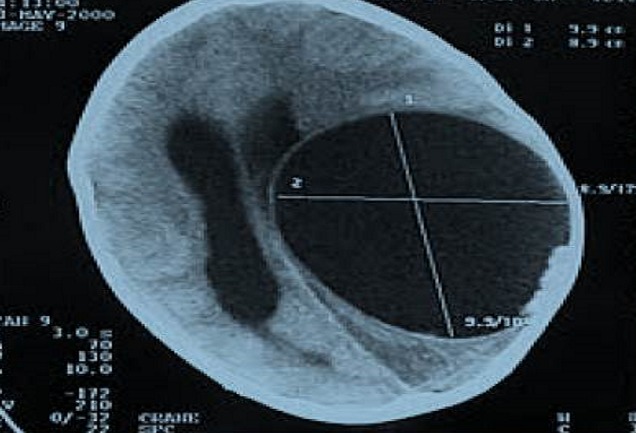
TDM d'un kyste hydatique cérébral de siège pariétal gauche

**Figure 3:**
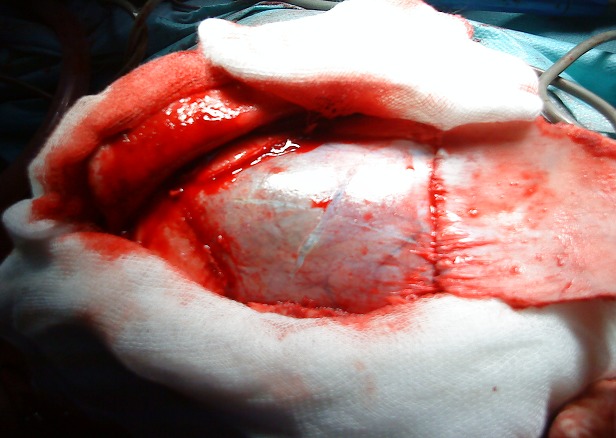
Craniotomie

**Figure 4:**
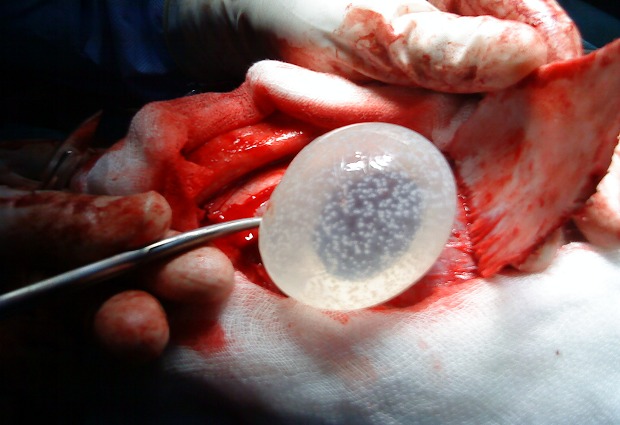
Accouchement du kyste

L’âge moyen était de 7 ans, avec des extrêmes allant de 5 à 11 ans, 4 garçons pour 1 fille, 3 patients étaient d'origine rurale et 2 d'origine urbaine.le délai de consultation était de 1 mois à 1 an avec une moyenne de 6,2 mois.les signes révélateurs étaient des signes d'hypertension intracrânienne dans 3 cas, des troubles de conscience:état comateux avec GCS à 4 dans un cas, des céphalées persistantes sans un réel syndrome d'HTIC dans un cas. La TDM cérébrale (Figure 1, Figure 2) a posé le diagnostic chez tout les malades, elle a objectivé une lésion unique avec un aspect radiologique typique: image hypodense bien limitée sans œdème prise de contraste en périphérie. Un bilan d'extension a été réalisé chez tous les malades, à la recherche de localisations autres notamment pulmonaire et hépatique: la radiographie du thorax et l’échographie hépatique et abdominale étaient normales. Le traitement était chirurgical dans tout les cas, avec craniotomie large, corticotomie (Figure 3), dissection hydrique et accouchement du kyste (Figure 4) selon la technique d'Arana Inguez. Un seul cas a été rompu en per-opératoire, pour lequel un traitement médical post-opératoire à base d'albendazol a été prescris pour prévenir la récidive. L’évolution a été marquée par le développement d'un accès maniaque chez un enfant, et d'une cécité définitive chez un autre, les trois autres patients ont bien évolué, sans récidive après un recul moyen de 1 an.

## Discussion

Le kyste hydatique cérébral est rare, il constitue 1 à 2% de toutes les localisations de l'organisme [3], et 2à 4% des processus non traumatiques intracérébraux, et touche l'enfant et l'adolescent dans plus de 60% des cas [2]. Cette rareté s'explique par l'existence de deux filtres: hépatique et pulmonaire qui empêchent l'arrivé du parasite au niveau du cerveau. Les signes clinques dépendent du siège du kyste, et de son volume.

L'hypertension intracrânienne est quasi constante [4], elle exprime la présence d'un processus expansif intracrânien qui s'installe de façon progressive sur plusieurs semaines voir plusieurs mois, du fait de la croissance lente du kyste et de l’élasticité relative de la boite crânienne de l'enfant: les céphalées sont progressives d'intensité croissante, les vomissements matinaux, soulageant les céphalées, Les troubles visuels sont difficiles parfois à mettre en évidence chez l'enfant jeune mais l’œdème papillaire uni ou bilatérale sont souvent retrouvés lors de l'examen ophtalmologique. Un seul de nos malades a présenté une baisse importante de l'acuité visuelle, et chez qui l'examen ophtalmologique a retrouvé un œdème papillaire. Chez les autres l'examen ophtalmologique n'a pas été fait. D'autres signes peuvent accompagnés ou révélés le kyste hydatique cérébral: déficit neurologique a type de parésie, ou rarement paralysie [5]; des crises convulsives tonicocloniques généralisés, surtout dans les formes remaniées ou calcifiés [6]; des troubles de comportements ou des troubles psychiatriques peuvent être présents voir même des troubles de consciences allant d'une obnubilation à un vrai coma [7].

La TDM cérébrale est l'examen de choix pour faire le diagnostic [8, 9], elle permet d'objectiver la lésion, de préciser le nombre, le contenu, le siège et surtout les caractéristiques: Image kystique hypodense, en plein parenchyme de même densité que le LCR, à paroi fine, refoulant le parenchyme et la ligne médiane, sans prise de contraste ni d’œdème perilésionnel. Bien que la TDM permet d'asseoir le diagnostic dans la majorité des cas [9], L'IRM est plus précise, elle montre le kyste hydatique cérébral sous forme d'une image liquidienne a paroi fine, avec les mêmes caractéristiques que le LCR: hypo intense en T1 et hyper intense en T2.l'absence de prise de contraste en périphérie est plus nette, et les rapports avec les structures avoisinantes sont mieux étudiés. L'IRM permet également une meilleure étude des kystes hydatiques multiples, des cas atypiques ou compliqués [6, 8]. L'IRM n'a été demandée chez aucun de nos malades, puisque l'aspect radiologique était typique à la TDM.

Le bilan biologique n'a pas un grand intérêt diagnostique, la sérologie hydatique qui est positive dans les kystes hépatiques dans 70% des cas ne l'est que dans 20% en cas de kyste hydatique cérébral isolé. Chez tous nos malades la sérologie hydatique était négative.

Le bilan d'extension recherche une localisation hépatique et/ou pulmonaire associée, ils sont les deux organes les plus touchés par la maladie hydatique. Ce bilan était négatif chez tout nos malades, ceci concorde avec les résultats de la littérature: le kyste hydatique cérébral de l'enfant est souvent primitif.

Le diagnostic différentiel peut se poser avec certaines lésions à composante kystiques, en particulier le kyste arachnoïdien, leptoméningé, épidermoïde, la cavité porencéphalique, l'astrocytome kystique, le craniopharyngiome et l'abcès du cerveau [5, 10]. Mais avec une bonne analyse radiologique, et le contexte épidémiologique, le diagnostic de kyste hydatique est souvent aisé.

Le traitement du kyste hydatique cérébral est chirurgical.et consiste à enlever le kyste sans le rompre. Par une craniotomie large, le kyste est disséqué par injection de sérum salé autour, le décollement du kyste se fait progressivement jusqu'a être accouché: c'est la technique d'Arana Inguiez.la ponction-aspiration du kyste est utilisé rarement dans les kystes de siège très profond [11].

La technique d’ Arana Inguiez été utilisé chez tous nos malades, on déplore une seule rupture en per-opératoire d'un volumineux kyste. Un cas de notre série a gardé une cécité définitive vu que la souffrance optique était irréversible. Un autre cas a développé un syndrome maniaque [12] après traitement du kyste hydatique. Il a été stabilisé sous traitement anti psychotique.

Le traitement médical à base d'albendazol est indiqué en cas de rupture, ou en cas de localisations multiples. Un malade de notre série a été mis sous albendazol à cause de la rupture per opératoire du kyste, il n'a pas présenté de récidives après un recul de 1 an.

## Conclusion

Le kyste hydatique cérébral est rare, il touche l'enfant et l'adolescent, les signes cliniques sont, dominés par l'hypertension intracrânienne. Le diagnostic est radiologique fait par la TDM. Le traitement est chirurgical avec un bon pronostic cependant la surveillance s'impose vu que le risque de récidive n'est pas négligeable.
